# DC-SIGN signalling induced by *Trichinella spiralis* products contributes to the tolerogenic signatures of human dendritic cells

**DOI:** 10.1038/s41598-020-77497-x

**Published:** 2020-11-20

**Authors:** Jelena Cvetkovic, Nataša Ilic, Alisa Gruden-Movsesijan, Sergej Tomic, Ninoslav Mitic, Elena Pinelli, Ljiljana Sofronic-Milosavljevic

**Affiliations:** 1 Institute for the Application of Nuclear Energy, University of Belgrade, Belgrade, Serbia; 2 Centre for Infectious Disease Control Netherlands, National Institute for Public Health and the Environment (RIVM), Bilthoven, The Netherlands

**Keywords:** Cell biology, Immunology

## Abstract

Tolerogenic dendritic cells (tolDCs) are central players in the maintenance of immune tolerance and thereby have been identified as the most favourable candidates for cell therapy of autoimmune diseases. We have recently shown that excretory-secretory products (ES L1) released by *Trichinella spiralis* larvae induce stable human tolDCs in vitro via Toll-like receptor 2 (TLR2) and TLR4. However, engagement of these receptors did not fully explain the tolerogenic profile of DCs. Here, we observed for the first time that dendritic cell-specific ICAM-3 grabbing non-integrin (DC-SIGN) interacts with highly glycosylated ES L1 and contributes to the generation of ES L1-induced tolDCs. Blocking DC-SIGN interfered with the ES L1-induced higher expression of CD40 and CCR7 and the production of IL-10 and TGF-β by DCs. The cooperation of TLR2, TLR4 and DC-SIGN receptors is of importance for the capacity of DCs to prime T cell response toward Th2 and to induce expansion of CD4+CD25+Foxp3+ T cells, as well as for the production of IL-10 and TGF-β by these cells. Overall, these results indicate that induction of tolDCs by ES L1 involves engagement of multiple pattern recognition receptors namely, TLR2, TLR4 and DC-SIGN.

## Introduction

*Trichinella spiralis* (*T. spiralis*), the parasitic nematode distributed throughout the world, causes chronic infection in a wide range of mammalian hosts including humans. After ingestion of raw or not fully cooked meat, the *T*. *spiralis* larvae migrate to striated muscles of the human host where it can remain encapsulated for many years. During the muscle stage of infection host-parasite interactions are established in such a way that generally nor the host or the parasite are harmed. To achieve this, the parasite has developed sophisticated survival strategies including the ability to modulate the host’s immune response^1^. Although many studies had reported a putative beneficial effect of *T. spiralis* on inflammatory diseases including ulcerative colitis^2^, autoimmune encephalomyelitis—EAE^3–5^, type 1 diabetes^6^, and allergic disorders^7^, it is clear that the use of live parasite to treat these diseases poses a health risk as well as an ethical issue that needs to be avoided. Therefore, the ultimate goal would be to understand the molecular mechanisms underlying immunomodulation as well as identifying the parasite’s molecules involve in this process, in order to make use of them for a putative immunotherapy of chronic inflammatory diseases. In the search for molecules and mechanisms involved in immunomodulation, special attention has been given to the excretory-secretory products (ES L1) released by *T. spiralis* muscle larvae. During the long-lasting muscle phase of the infection, ES L1 continuously stimulates host cells and modulates the host immune responses^8–11^. These products depict around forty different macromolecules that exhibit a broad range of biological activities^12^. It has been shown in animal models that these products induce semi mature/tolerogenic status of dendritic cells (DCs) that shift the immune response towards a Th2 and regulatory type of T cells^10,11^. Furthermore, the use of ES L1-exposed DCs or isolated ES L1 products could, in a great deal, mimic the immune response of actual *T. spiralis* infection and alleviate EAE in rat animal model^5,13^. Even though the ability of ES L1 products to induce tolDCs has been delineated and has successfully ameliorated autoimmunity in animal models^4^, cell therapies based on ES L1 products require extensive studies on a human DC model system. Our study has recently revealed that ES L1 treatment of human monocyte-derived DCs from healthy donors leads to a stable tolerogenic phenotype of DCs, the cells which have the potential to induce Th2 and a regulatory type of immune response^14^. These results are promising and have considerable potential in helping with the design of new cell-based tolerance-inducing therapies for inflammatory diseases. However, further studies toward uncovering the molecular basis of tolerogenic features of ES L1-induced DCs are necessary. Hitherto, it has been revealed that Toll-like receptor 2 (TLR2) and TLR4 bind ES L1 products and are important for the ES L1-induced tolerogenic status of human DCs^14^. Although the TLRs, especially TLR4, has so far been widely associated with the initiation of Th1 inflammatory but not Th2 responses, it has been shown that some helminth molecules are involved in signalling via several pattern recognition receptors (PRRs)^15^. One possible explanation for the mechanism by which helminth products divert pro-inflammatory TLR signalling into anti-inflammatory responses is through TLR cooperation with other PRRs, such as C-type lectin receptors (CLRs). CLRs on DCs sense the carbohydrate structures displayed on different pathogens, participate in their internalization, degradation, presentation and lead to activation of the signalling cascade which in turn leads to the activation of DCs and consequent polarization of the T cell response^16–18^. These receptors activate DCs by inducing gene expression independently of other PRRs, or by modulating TLR-induced gene expression at the transcriptional or posttranscriptional level^19–23^. Several studies have shown that the mannose receptor (MR), macrophage-galactose-type lectin (MGL) and DC-specific ICAM-3-grabbing non-integrin (DC-SIGN) on human DCs recognize glycans from soluble egg components of *Schistosoma mansoni* (*S. mansoni*), *Trichuris suis* (*T. suis*), *Ascaris suum* (*A. suum*) or *Fasciola hepatica* (*F. hepatica*)^24–28^. Additionally, *F. hepatica* glycoconjugates via DC-SIGN on DCs suppress allogeneic T cell proliferation and drives T regulatory immune response^28^. ES L1 products of *T. spiralis* muscle larvae are highly glycosylated^29^, decorated with high-mannose type residues and as such good candidates for CLR ligands. Sugar residues are crucial for the function of ES L1 products, since their chemical modification abolished binding to macrophage MR^30^ and impeded immune response polarization toward Th2 and regulatory type^31^. The tolerizing effect of ES L1 on the phenotype and the function of DCs could be explained by its binding to DC-SIGN that ultimately leads to a modulation of TLR-triggered signalling. However, the interaction of the ES L1 products with DC-SIGN receptor, abundantly expressed on DCs^16,32^ has not been explored yet. In this study, we hypothesize that ES L1 interacts with DC-SIGN and that engagement of this PRR by these helminth products is necessary for the efficient induction of the tolerogenic properties of human monocyte derived DCs.

## Results

### DC-SIGN binds *Trichinella spiralis* ES L1 products

**Figure 1:**
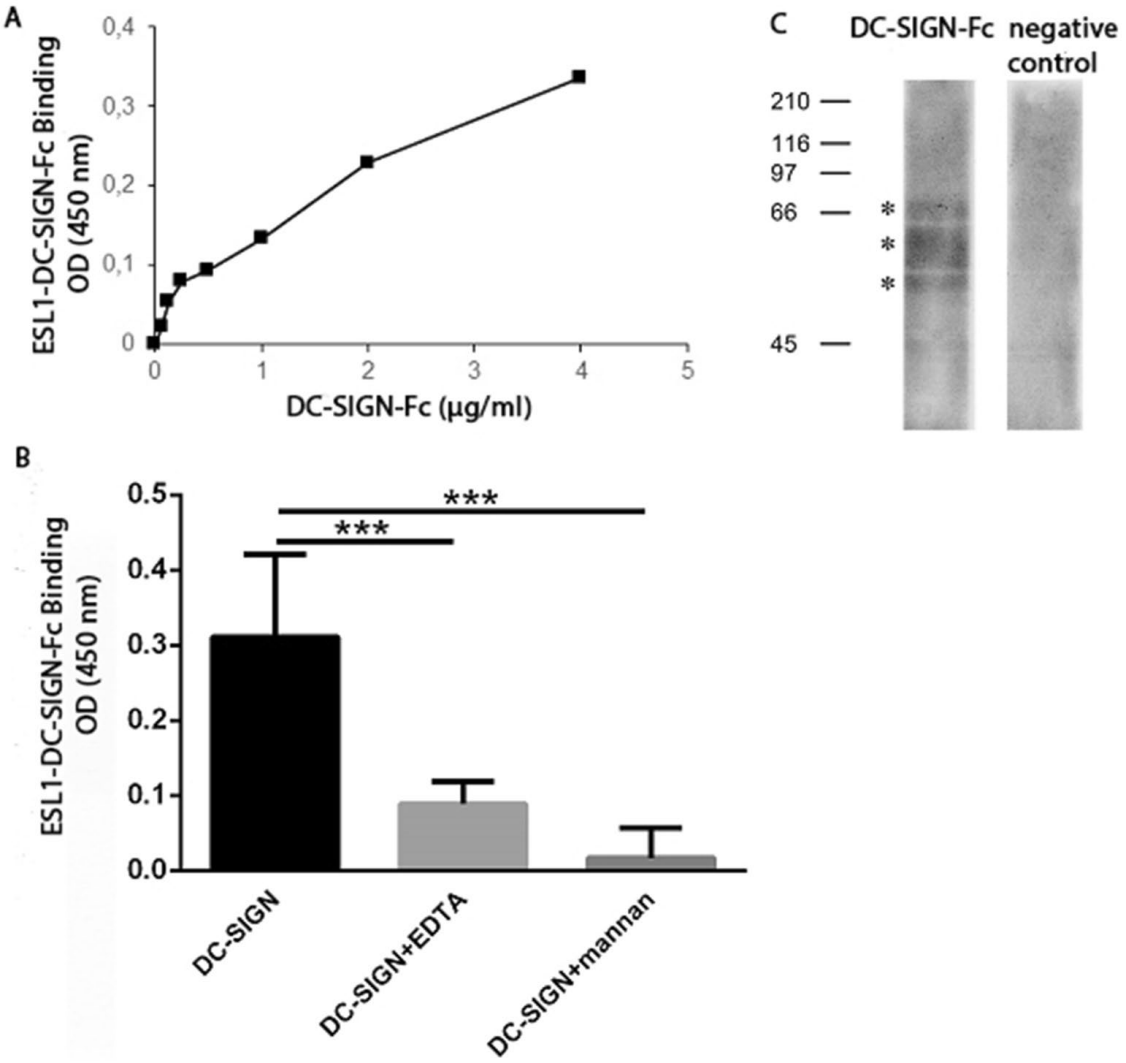
Binding of DC-SIGN-Fc chimera protein to immobilized ES L1 products. (**A**) Dose dependent manner of DC-SIGN-Fc binding to microwell plate coated with ES L1. (**B**) The specificity of DC-SIGN-Fc binding to ES L1 demonstrated by the inhibition of the reaction with EDTA and mannan. The results are presented as mean values of two independent experiments. Differences were analysed for significance by one-way ANOVA with the Bonferroni’s multiple comparison test: ***p < 0.001. (**C**) DC-SIGN-Fc recognizes 49, 53 and 66 kDa ES L1 glycoproteins in Western blot.

Several studies have shown the importance of the DC-SIGN receptor in binding helminth antigens^33,34^, but there is no information yet on the role of this receptor in *T. spiralis* antigen binding. The interaction of DC-SIGN with ES L1 products was investigated using a chimera construct of DC-SIGN and human IgG1 Fc fragment in a binding assay and Western blot, following detection with biotinylated mouse anti-human Fc-specific IgG. DC-SIGN-Fc chimera protein specifically reacted with ES L1, immobilized on microwell plates, in a dose-dependent manner (Fig. 1A). This interaction was mediated by the carbohydrate recognition domain of DC-SIGN, since it was inhibited with mannan, a specific ligand for this receptor and with EDTA which removes Ca^2+^ ions that are essential for the activity of carbohydrate recognition domain of DC-SIGN^35^ (Fig. 1B). The inhibition of binding was 57.4% in the case of EDTA and 94.5% when mannan was used. In Western blot, it was observed that DC-SIGN-Fc specifically recognized three ES L1 glycoproteins at 49, 53 and 66 kDa. Cropped, relevant section of the blot is shown for clarity (Fig. 1C), with the full length blot included in Supplementary Information, Fig S1.

### Blocking DC SIGN hinders a tolerogenic DC phenotype induced by ES L1

Our previous findings implicated TLR2 and TLR4 in ES L1 induction of tolDCs^14^. Aforementioned results demonstrated that DC-SIGN also binds ES L1. The next step was to investigate the role of DC-SIGN in ES L1 driven tolerization of DCs.

**Figure 2:**
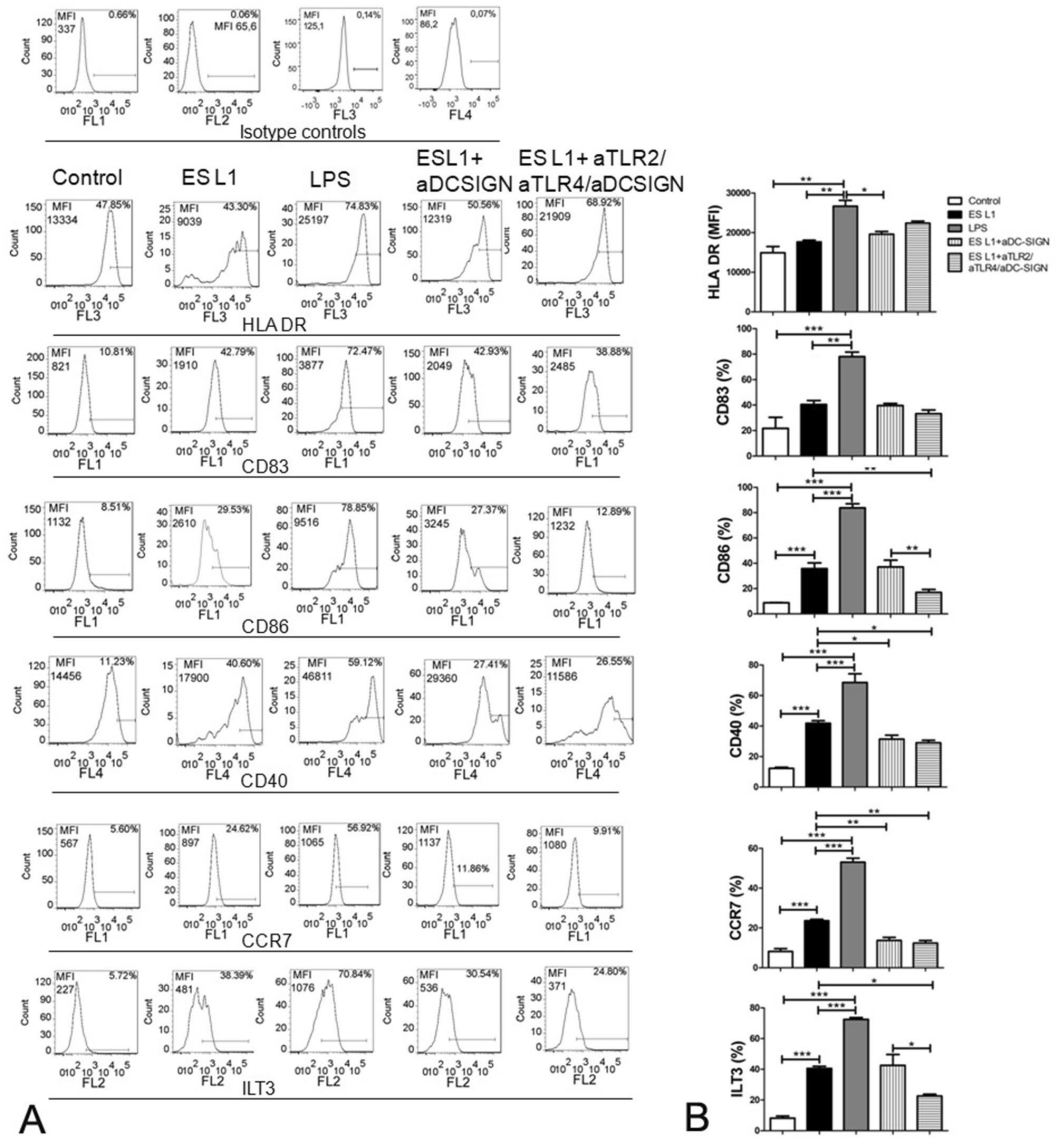
Effect of DC-SIGN, TLR2 and TLR4 blocking on the expression of maturation markers in ES L1 treated dendritic cells (DCs). Immature DCs were incubated with ES L1 (50 μg/ml) alone, as well as in the presence of specific DC-SIGN blocking antibodies (20 ng/ml) alone or simultaneously with TLR2 and TLR4 blocking antibodies (10 μg/ml). Non-treated cells cultivated in medium were used as a negative control (control) and LPS (200 ng/ml) treated DCs were used as a positive control for complete maturation. After 48 h, the expression of markers (HLA-DR, CD83, CD86, CD40, CCR7 and ILT3) was measured by flow cytometry. (**A**) Representative analysis of surface markers expression is shown, and (**B**) the summarized results are shown as mean value of percentage (%) of markers expression ± SD from three different experiments. *p < 0.05, **p < 0.01, ***p < 0.005 as indicated by line (one-way ANOVA with Tukey’s posttest).

Differentiated immature DCs were more than 85% CD1a positive (see Supplementary Information, Fig. S2). These cells were incubated with blocking antibody against DC-SIGN alone, or simultaneously with blocking antibodies against TLR2 and TLR4 receptors before treatment with ES L1 products, and the phenotype of cells was examined. Obtained results for the effect of ES L1 on the expression of surface markers and cytokine production of DCs are in accordance with previous findings^14^ indicating the induction of a tolerogenic phenotype. Namely, ES L1 did not affect the expression of HLA-DR, but it induced significantly higher expression of CD86, CD40, CCR7 and immunologlobulin like transcript 3 (ILT3), marker of tolerogenic DCs, compared to control cells, cultivated in medium only (Fig. 2). DC-SIGN neutralization did not disrupt the expression of most of the surface markers, except for CD40 and CCR7. Simultaneous blocking of all three receptors showed comparable effect as with the DC-SIGN blocking indicating the importance of DC-SIGN but not TLR2 and TLR4 in the expression of these surface markers induced by ES L1. On the other hand, simultaneous blocking highlights the importance of TLR2 and TLR4 engagement for the expression of CD86 (Fig. 2, Supplementary Fig. S3A).

**Figure 3:**
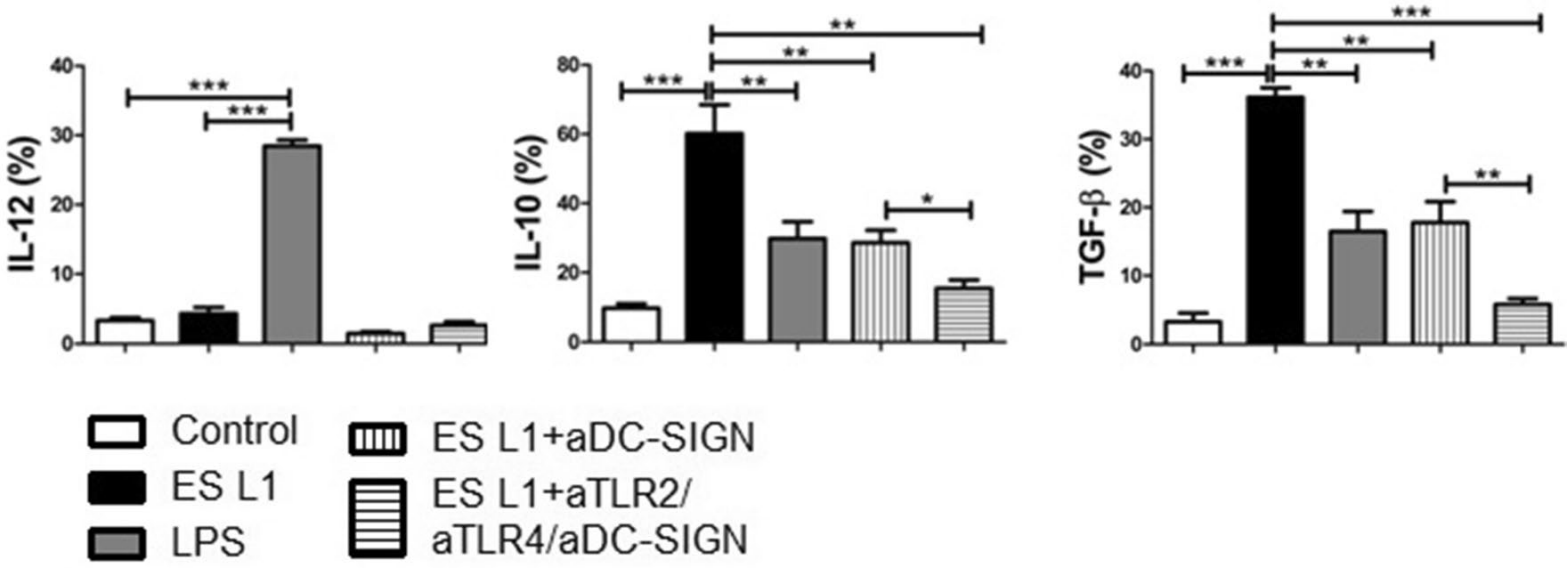
Effect of DC-SIGN, TLR2 and TLR4 blocking on the cytokine expression in ES L1-treated dendritic cells (DCs). The analysis of interleukin (IL)-10, IL-12 and transforming growth factor (TGF)-β expression within DCs was performed by intracellular staining, measured by flow cytometry and shown as mean ± SD of the percentage (%) of cytokines expression from three different experiments. *p < 0.05, **p < 0.01, ***p < 0.005 compared to control (non-treated cells cultivated in medium), or as indicated (one-way ANOVA with Tukey’s posttest).

ES L1 did not affect the production of proinflammatory cytokine IL-12, while it stimulated significant production of IL-10 and TGF-β (Fig. 3). Blocking experiments revealed that engagement of DC-SIGN by ES L1 is of a great importance for the induction of IL-10 and TFG-β production. Blocking of all three receptors, DC-SIGN, TLR2 and TLR4, induced even more profound decrease in the percentage of IL-10+ and TGF-β+ DCs compared to the effect exerted by individual DC-SIGN blocking, suggesting that cumulative effect of all three receptors is crucial for the expression of these cytokines triggered by ES L1 (Fig. 3, Supplementary Fig. S3B).

**Figure 4:**
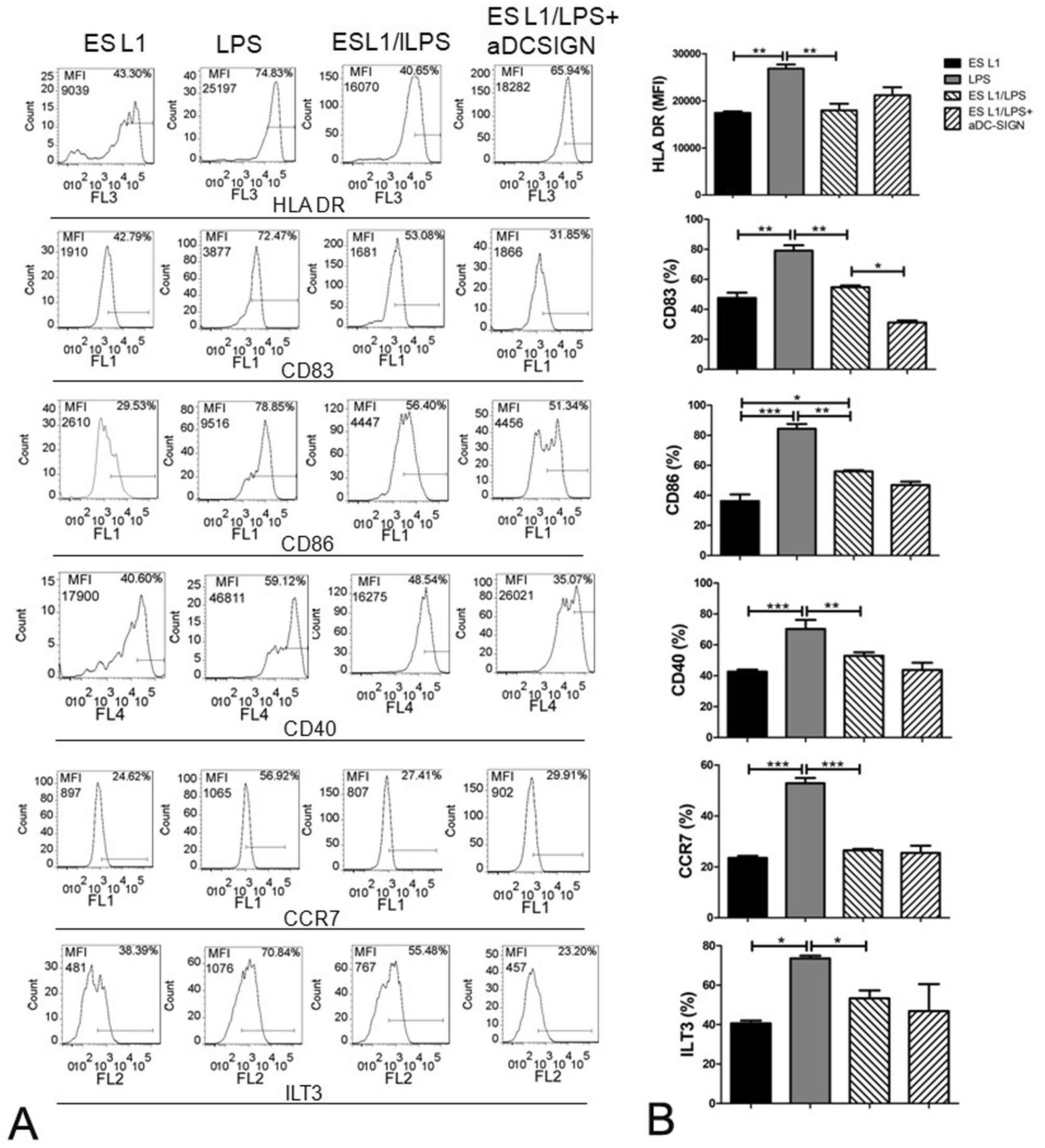
Effect of DC-SIGN blocking on the expression of maturation markers in ES L1 treated dendritic cells (DCs). Immature DCs were treated with ES L1 products (50 μg/ml), in the presence or in the absence of specific DC-SIGN blocking antibodies (20 ng/ml), on day 4 of culture for 24 h and then additionally activated or not with LPS for the next 24 h, followed by flow cytometry analysis. (**A**) Representative analysis of the surface markers expression is shown) (gating strategy is presented in Fig. 2), and (**B**) the summarized results of percentages of HLA-DR, CD83, CD86, CD40, CCR7 and ILT3 expression by DCs are shown as mean ± SD from three different experiments. *p < 0.05, **p < 0.01, ***p < 0.005 compared as indicated (one-way ANOVA with Tukey’s posttest).

**Figure 5:**
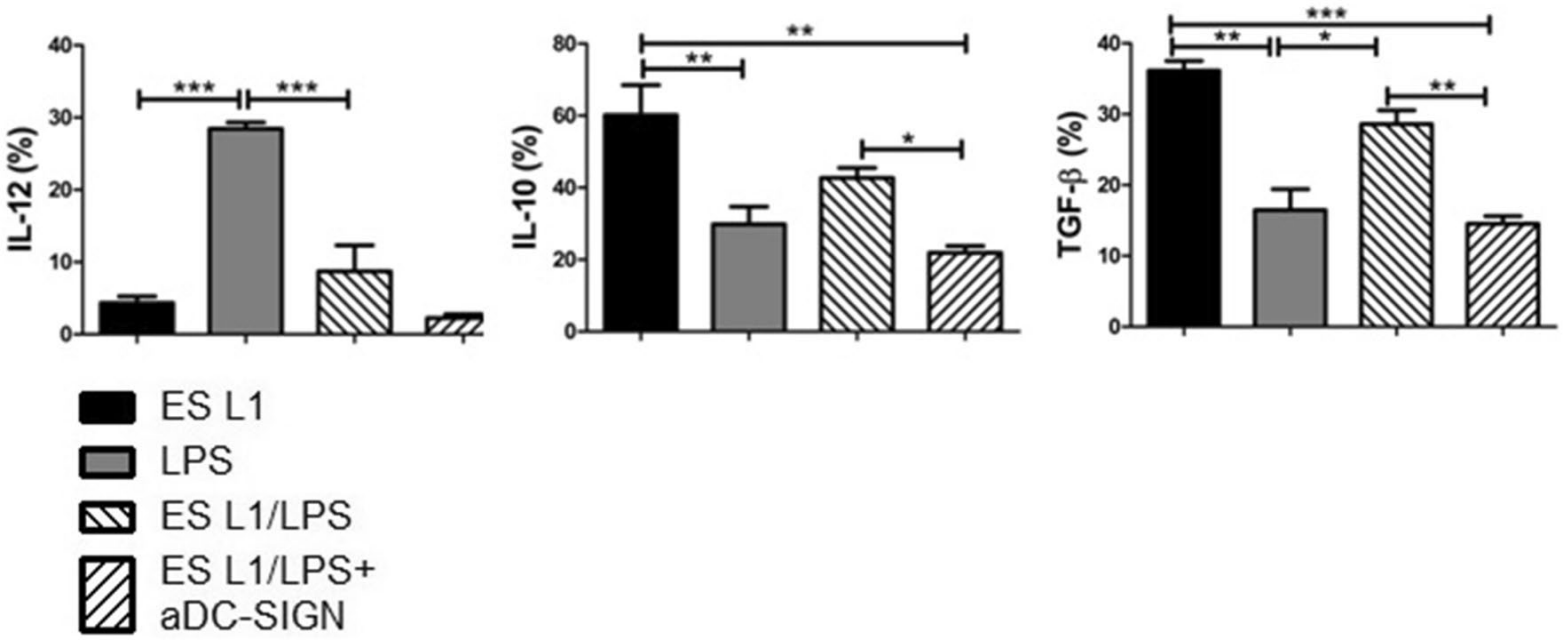
Effect of DC-SIGN blocking on the cytokine expression in ES L1-treated dendritic cells (DCs) challenged with inflammatory stimulus (LPS, 500 ng/ml). The analysis of interleukin (IL)-10, IL-12 and transforming growth factor (TGF)-β expression within DCs was performed by intracellular staining, measured by flow cytometry and shown as mean ± SD of the percentage (%) of cytokines expression from three different experiments. *p < 0.05, **p < 0.01, ***p < 0.005 compared as indicated (one-way ANOVA with Tukey’s posttest).

As expected, LPS induced full DC maturation as defined by significantly higher expression of all examined maturation markers HLA-DR, CD83, CD86, CD40, CCR7 and ILT3. To challenge the stability of tolerogenic ES L1-induced DCs, LPS was added to cell cultures 24 h after ES L1 stimulation. ES L1 treated cells retained the tolerogenic phenotype and function despite inflammatory stimulus (Figs. 4 and 5). Blocking of DC-SIGN affected the maintenance of elevated IL-10 and TGF-β production in ES L1 treated DCs after LPS challenge, which indicated that DC-SIGN participate in the induction of a stable ES L1-induced tolerogenic phenotype of DC that is not altered by additional TLR4 activation (Fig. 5).

### ES L1-induced anti-inflammatory and regulatory responses are dependent on the engagement of DC-SIGN

**Figure 6:**
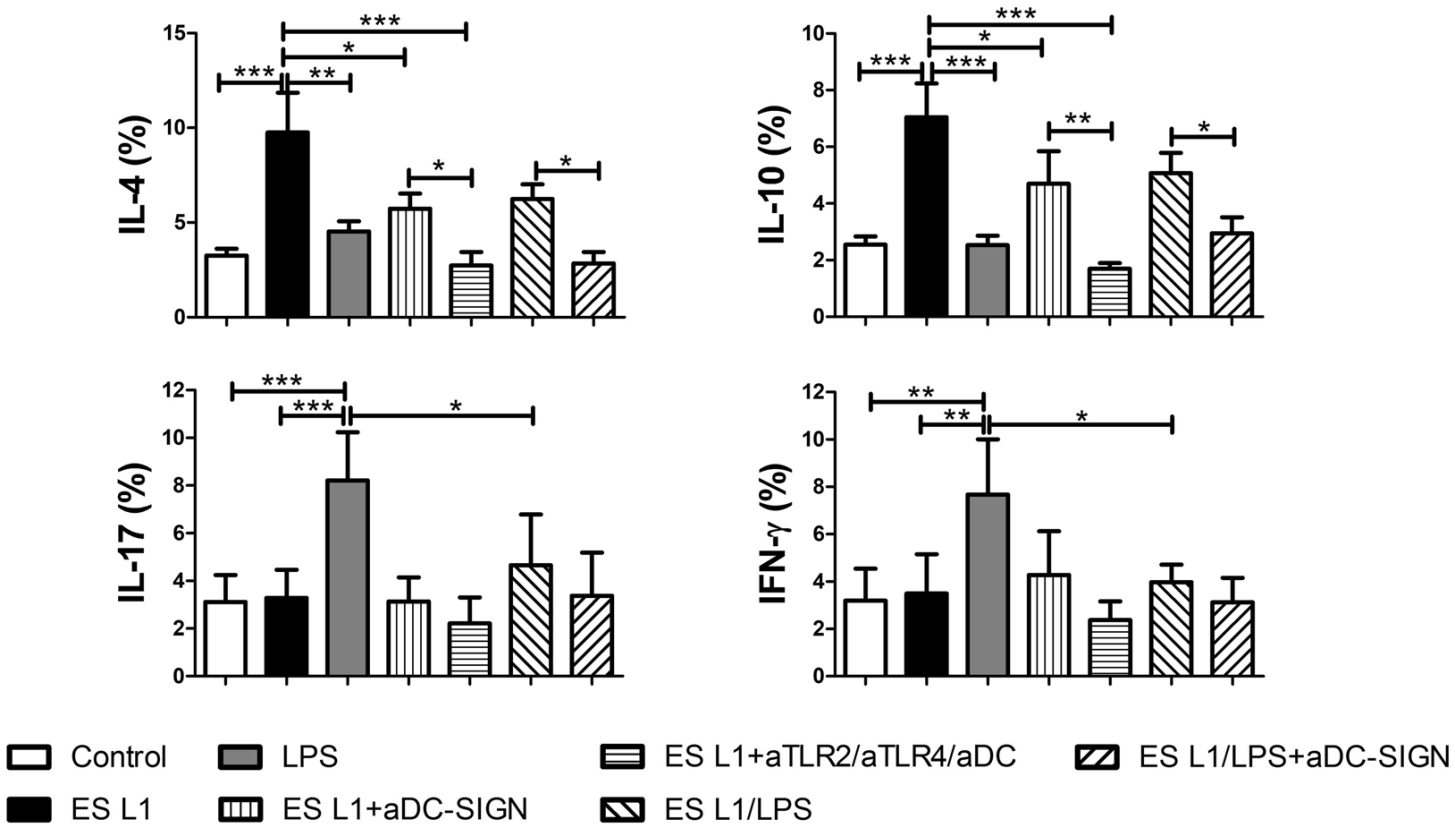
Impact of DC-SIGN on T helper polarization induced by ES L1-pulsed dendritic cells (DCs). DCs treated with specific DC-SIGN blocking antibodies, alone or simultaneously with TLR2 and TLR4 blocking antibodies, prior to ES L1 and/or LPS, were washed thoroughly and then cocultured with magnetic-activated cell sorting-purified allogenic T cells (Tly) (1 × 10^5^/well) for 6 days in 1:20 DC:T cell ratio. Upon cocultivation, the percentage of cytokines expression was measured intracellularly by flow cytometry, within the T cells subjected to CD4 surface staining prior to intracellular staining, and treated with PMA/Ionophore/monensin for the last 4 h. The summarized results are shown as mean percentages (%) ± SD of three experiments with different DCs donors. *p < 0.05, **p < 0.01, ***p < 0.001 compared to control (non-treated cells cultivated in medium), or as indicated (one-way ANOVA with Tukey’s posttest).

To investigate DC-SIGN as a possible candidate receptor on DCs involved in the ES-L1-induced Th2 and a regulatory type of immune response, human monocyte derived DCs, pre-incubated with DC-SIGN, TLR2 and TLR4 blocking antibodies before the treatment with ES L1, were cocultivated with allogeneic T cells. In line with our previous study^14^, ES L1 shapes immune response favouring Th2 and regulatory T cell polarization characterized by higher production of IL-4 and IL-10 but no IL-17 and IFN-γ, which production was at the level of untreated controls (Fig. 6, Supplementary Fig. S4). In order to investigate the importance of DC-SIGN signalling for the observed effects, we measured intracellular cytokines (IL-4, IL-10, IFN-γ and IL-17) in T cells after their co-cultivation with ES L1-treated DCs either with or without previous DC-SIGN blocking. The obtained results revealed that the blocking of DC-SIGN receptor correlated with a lower production of IL-4 and IL-10 and that the production of these cytokines was totally abolished by simultaneous blocking of DC-SIGN, TLR2 and TLR4 prior to ES L1 treatment of DCs. ES L1 treated DCs were resistant to challenge with LPS, in terms of the percentage of IL-4+ , IL-10+ , IFN-γ+ and IL-17+ cells within CD4+ T cell population. Moreover, blocking of DC-SIGN before ES L1 stimulation abolished the effect of ES L1 on subsequent LPS challenge regarding IL-4+ and IL-10+ cells compared to the effect that this challenge had in ES L1 treated DCs without the receptors blocking (Fig. 6, Supplementary Fig. S4).

**Figure 7:**
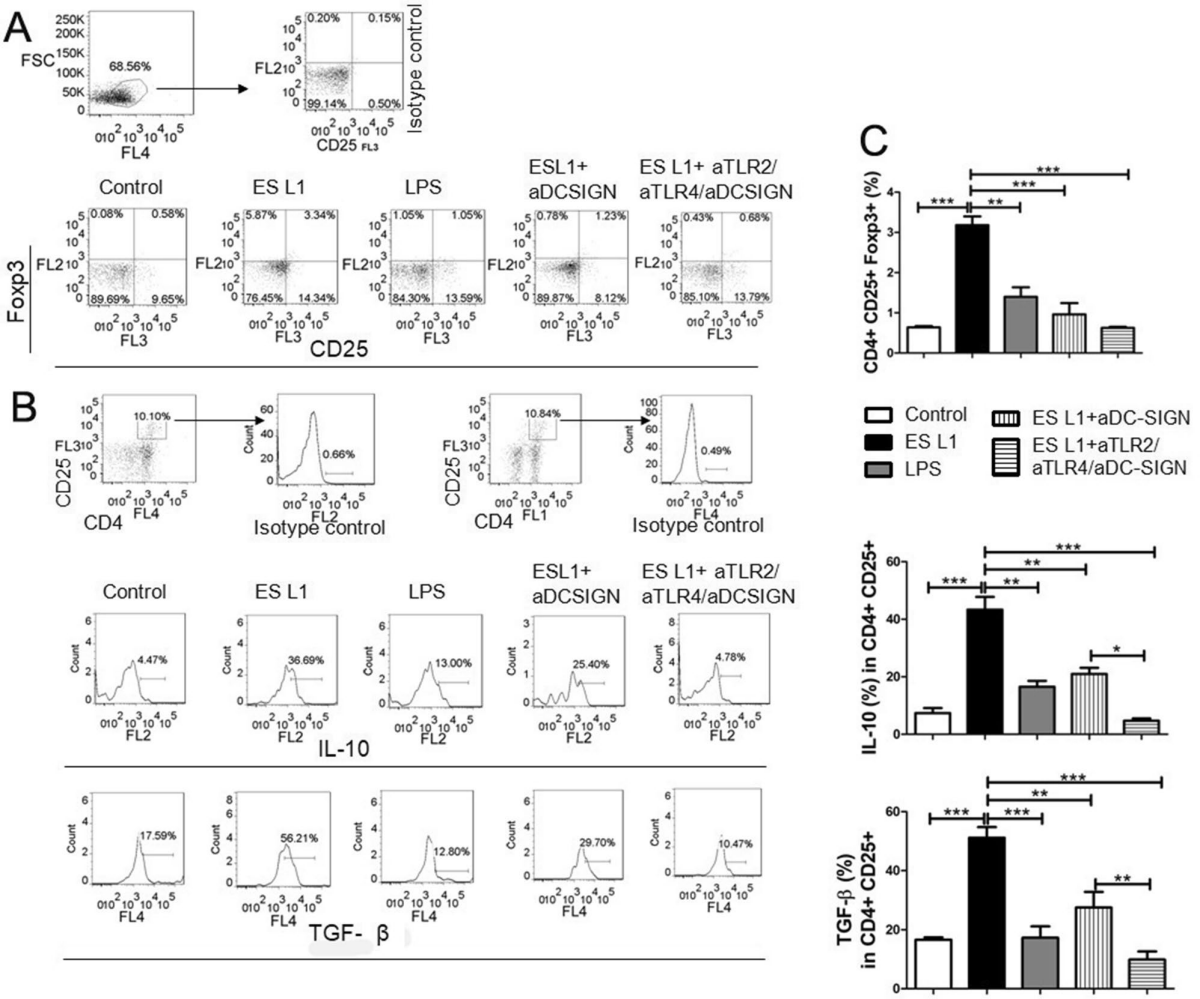
Impact of DC-SIGN on tolerogenic properties and functions of ES L1-treated dendritic cells (DCs). DCs treated with specific DC-SIGN blocking antibodies, alone or simultaneously with TLR2 and TLR4 blocking antibodies, prior to ES L1 products (50 μg/ml) were washed thoroughly and then cocultured with magnetic-activated cell sorting-purified allogenic T cells (Tly) (1 × 10^5^/well) for 3 days in 1:50 DC:T cell ratio and then re-stimulated with interleukin (IL)-2 (2 ng/ml) for another 3 days. (**A**) Representative analysis of CD25+FoxP3+ cells within CD4+ T cells from one experiment is shown, (**B**) Representative analysis of IL-10 and transforming growth factor (TGF)-β within CD4+ CD25+ T cell population is shown, and (**C**) the summarized results for the expression of CD4+CD25+FoxP3+ T regulatory cells and IL-10 and TGF-β within CD4+CD25+T cells are shown as the mean% ± SD from three different experiments. *p < 0.05, **p < 0.01, ***p < 0.005 compared to control (non-treated cells cultivated in medium), or as indicated (one-way ANOVA with Tukey’s posttest).

**Figure 8:**
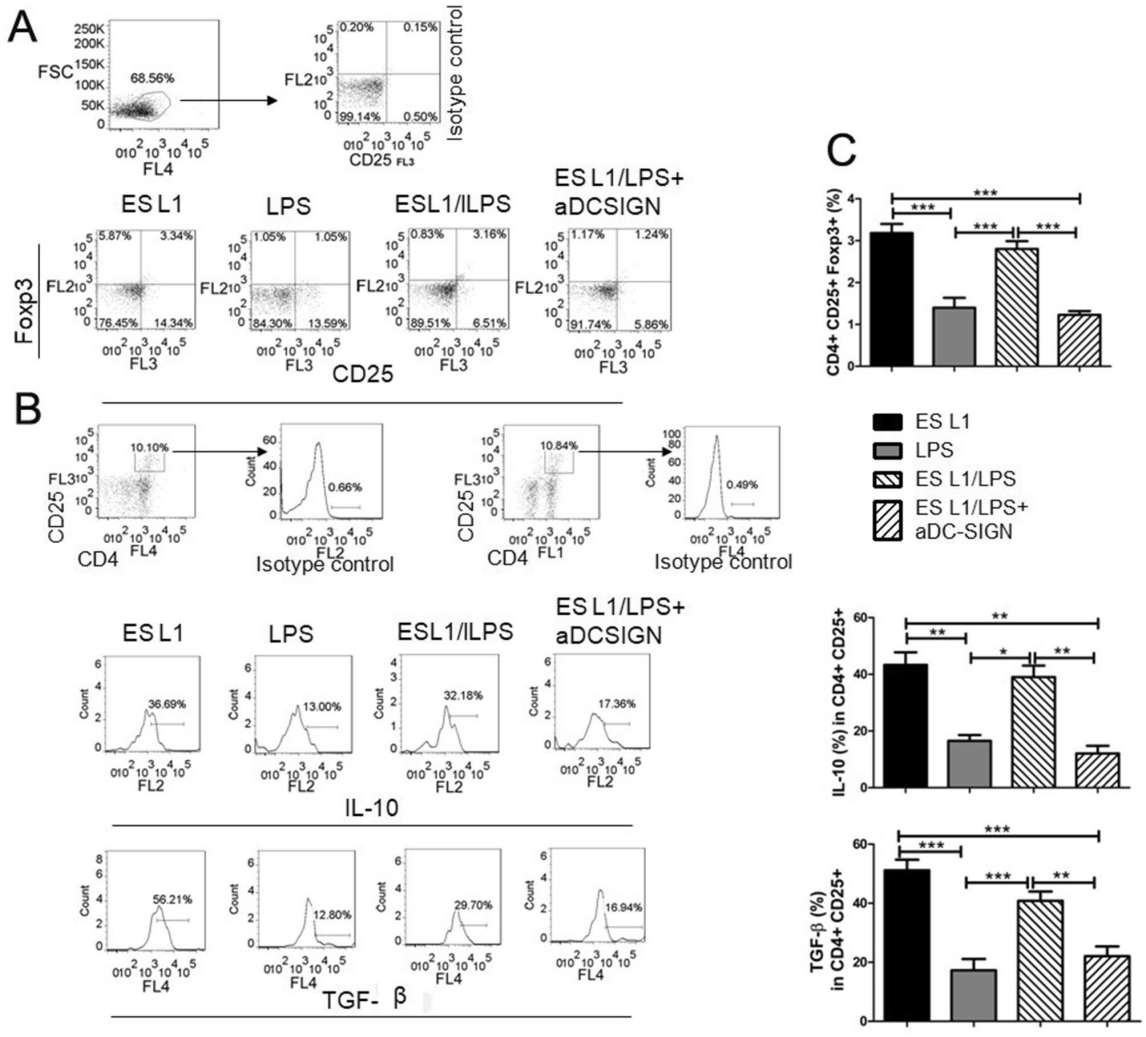
Impact of DC-SIGN on tolerogenic properties and functions of ES L1-treated dendritic cells (DCs) challenged with pro-inflammatory stimulus. DCs treated with ES L1 products (50 μg/ml), in the presence or in the absence of specific DC-SIGN blocking antibodies (20 ng/ml), and then additionally activated or not with LPS, were washed thoroughly and then cocultured with magnetic-activated cell sorting-purified allogenic T cells (Tly) in 1:50 DC:T cell ratio, as described. (**A**) Representative analysis of CD25+FoxP3+ cells within CD4+ T cells from one experiment is shown, (**B**) Representative analysis of IL-10 and transforming growth factor (TGF)-β within CD4+CD25+ T cell population is shown, and (**C**) the summarized results for the expression of CD4+CD25+FoxP3+ T regulatory cells and IL-10 and TGF-β within CD4+CD25+ T cells are shown as the mean% ± SD from three different experiments. *p < 0.05, **p < 0.01, ***p < 0.005 compared as indicated (one-way ANOVA with Tukey’s posttest).

To further evaluate whether ES L1 interaction with DC-SIGN is of importance for the generation of Tregs, allogeneic T cells were co-cultured with ES L1 stimulated DCs pretreated with DC-SIGN blocking antibodies. ES L1 significantly elevated the percentage of CD4+CD25+Foxp3+ cells (Fig. 7A,C), as well as the percentage of IL-10+ and TGF-β+CD4+CD25+ T cells (Fig. 7B,C). Neutralisation of DC-SIGN receptor significantly reduced the percentage of CD4+CD25+Foxp3+ Treg cells compared to ES L1 treated DCs. Moreover, the observed high level in the percentage of IL-10+ and TGF-β+CD4+CD25+Foxp3+ T cells induced by ES L1 treated DCs, was significantly decreased when DC-SIGN was blocked before ES L1 treatment of DCs. Additionally, simultaneous blocking of TLR2, TLR4 and DC-SIGN completely suppressed T cells polarization towards a Treg phenotype (Fig. 7, Supplementary Fig. S3C), highlighting the importance of the interaction of ES L1 with these receptors for ES L1 driven T regulatory immune responses. ES L1 stimulated DCs retained the capacity to induce high percentage of CD4+CD25+Foxp3+ cells, as well as the percentage of IL-10+ and TGF-β+CD4+CD25+Foxp3+ T cells even after LPS challenge (Fig. 8). DC-SIGN neutralisation abolished the tolerizing effect of ES L1 on DCs.

## Discussion

The results of this study show that DC-SIGN recognises and binds ES L1 products released by *Trichinella spiralis* muscle larvae. Foremost, it was demonstrated that this receptor, alongside TLR2 and TLR4, is a major contributor to ES L1 induced tolerogenic DC phenotype, which limits the production of pro-inflammatory cytokines by effector T cells and promotes Th2 and Treg polarization.

Next to TLRs, DC-SIGN plays an essential role in regulating DCs function by the recognition of a plethora of glycans exposed on multiple pathogens such as bacteria, viruses, fungi and parasites^32,36,37^. Here we examined the involvement of DC-SIGN in the binding of ES L1, since there was no information so far on the engagement of this receptor in the immune response mediated by ES L1 during *T. spiralis* infection. Our study shows that DC-SIGN recognizes and binds three ES L1 glycoproteins at 49, 53 and 66 kDa. These glycoproteins bare mannosylated and fucosylated residues^29^, which are specific ligands for DC-SIGN^38^. These results are consistent with the results of other studies which have demonstrated that DC-SIGN interacts with helminth glycoproteins, like *F. hepatica* glycoconjugates^28^ or *S. mansoni* SEA^21,39^. The interaction of DC-SIGN with ES L1 products was specific, since it was inhibited with EDTA and mannan. EDTA removes calcium necessary for DC-SIGN binding^35^, while mannan, as a specific ligand^40^, prevents binding to the carbohydrate recognition domain of DC-SIGN.

tolDCs are pivotal mediators of peripheral and central immune tolerance and because of their capacity to modulate immune response in antigen-specific manner represent tempting candidates for specific immunotherapy of autoimmune diseases such as multiple sclerosis, rheumatoid arthritis, type 1 diabetes etc^41–43^. In this study, human DCs treated with ES L1 acquired stable tolerogenic phenotype, characterized by low expression of co-stimulatory molecules and the production of significantly elevated concentrations of IL-10 and TGF-β. These results are in line with our previous finding that ES L1 renders tolerogenic human DCs, capable of inducing Th2 and regulatory responses in vitro^14^. Still, the molecular mechanisms governing ES L1 driven tolerogenic phenotype and functionality of DCs have yet to be fully portrayed. It is well known that DCs sense of pathogen associated molecular patterns (PAMPs) through PRRs initiates the cascade of signalling, leading to the appropriate immune response^44^. Regarding *T. spiralis* products, previous work has demonstrated that ES L1-induced tolerogenic properties of DCs involves TLR2 and TLR4 activation^14^, which was surprising, as these receptors have been mostly associated with PAMPs inducing dominant Th1 responses^45^. However, several studies have highlighted that the signalling pathways triggered in DCs by TLR ligation with helminth molecules, differ from that induced by LPS^46–48^. The differences between downstream signalling pathways provoked by TLR4 ligation with LPS and ES L1 may be the consequence of the different stimuli involved, but also by the engagement of other PRRs, such as CLR^17^. DC-SIGN is a C-type lectin receptor which is expressed to a high level on DCs. DC-SIGN activation does not induce the cytokine expression itself, but it adjusts signalling cascades triggered by other PRRs and thus determines the fate of the immune response^21^. It was reported that activation of DC-SIGN can affect the transition of inflammatory to anti-inflammatory phenotype of DC and alter the Th1/Th2 ratio^16,25,49^. In line with this observation Klaver et al.^25^ have delineated that *T. suis* glycans, via DC-SIGN, modulate the immune response of human DCs and attenuate LPS-induced expression of pro-inflammatory cytokines (TNF-a, IL-6, IL-12). Quite the opposite function was described in the case of *S. mansoni* worm glycolipids, where it was found that DC-SIGN is required for TLR4-induced activation of inflammatory phenotype of human DCs^34^. Our work highlighted the role of DC-SIGN in the induction of a tolerogenic phenotype of human DCs, provoked by ES L1. We have already demonstrated that glycans present on ES L1 products are indispensable for the induction of Th2 and anti-inflammatory responses (IL-4 and IL-10 production)^31^. ES L1, via binding to DC-SIGN, induced expression of CD40 and CCR7 on human DCs, which are characteristic for Th2-polarizing^50^ and tolerogenic DC^51^, respectively. Additionally, ES L1 induces increased production of key immunoregulatory cytokines IL-10 and TGF-β which was also DC-SIGN dependent. Namely, blocking of DC-SIGN before DC treatment with ES L1 led to decreased expression of afore mentioned surface markers and cytokines, even after restimulation with LPS. Similar role of DC-SIGN was described in the case of *F. hepatica* infection. The receptor was implicated in the induction of tolerogenic DCs, hence in installing an immunoregulatory environment^28^. Our previous work has shown that TLR2 and TLR4 are involved in the tolerizing effect that ES L1 exert on DC phenotype^14^. Simultaneous blocking of TLR2, TLR4 and DC-SIGN revealed the importance of the engagement of all three receptors for ES L1-induced IL-10 and TGF-β production in DCs. Interleukin-10 and TGF-β produced by DCs provide negative signalling to T cells and repress the production of pro-inflammatory cytokines, while at the same time promote Th2 and regulatory immune responses^52,53^. This type of immune response controls the excessive inflammation and creates an unfavourable environment for the development of autoimmune diseases. IL-12 represents a link between innate and adaptive immunity, being a mediator of the early innate immune response and has influence on the Th1 differentiation^54^. In this study, the low level of IL-12p70 production is likely due to increased IL-10 and TGF-β production by DC. This is supported by the work of Du and Sriram^55^, which showed that IL-10 and TGF-β suppress IL-12 production by inhibiting the transcription of IL-12p40 gene. Also, studies show that the IL-10/IL-12 ratio affects the polarization of CD4+ T lymphocytes^56,57^, in this case it drives immune response towards Th2 and regulatory type. Although the mechanisms of the cross-talk among DC-SIGN, TLR2 and TLR4 have not been investigated yet, we assume that DC-SIGN signalling pathways interfere with TLR-induced signalling, as delineated for other pathogens. It was described that the DC-SIGN signalling provoked by pathogens, such as *Mycobacterium tuberculosis* and *Candida albicans*, converges the TLR signalling pathways via the serine-threonine protein kinase c-RAF. This enzyme, activated by DC-SIGN, acetylates NF-κB subunit p65, but only if NF-kB is previously activated by TLRs. Acetylation of p65 leads to increased IL-10 gene expression^36^. Also, Terrazas and colleagues^23^ have reported that silencing of c-RAF in DCs treated with *Taenia crassiceps* antigens diminished the capacity of antigens to induce production of anti-inflammatory cytokines by DCs and promote Th2 response.

DCs orchestrate T cell polarization via cytokine production and expression of co-stimulatory molecules. Our previous work showed that ES L1 prime human DCs for Th2 and T regulatory polarization^14^. The present study shows that ES L1-treated DCs induce naïve allogeneic CD4+ T cells to produce high levels of IL-4, IL-10, TGF-β but no IL-17 and IFN-γ. Blocking of DC-SIGN on DCs diminished the potential of ES L1 to polarize T cells producing Th2 and anti-inflammatory type cytokines. Foremost, simultaneous blocking of DC-SIGN, TLR2 and TLR4 on DCs resulted in the inhibition of IL-4 and IL-10 production by T cells. This finding indicated that, besides already reported role of TLR2 and TLR4 in the induction of tolerogenic DC phenotype by ES L1, the interaction of ES L1 with DC-SIGN is very important for the capacity of DCs to polarize the immune response towards Th2 and anti-inflammatory type of immune responses, and also implicate the cumulative effect of these receptors in ES L1 in the production of IL-4 and IL-10. The polarizing capacity of ES L1-treated DCs was retained under inflammatory stimuli and engagement of DC-SIGN was shown to be crucial. Our findings are in agreement with the results from studies involving other helminths, like *A. suum* and *S. mansoni*. It was demonstrated that *A. suum* components containing N-linked oligosaccharides, via DC-SIGN and MR, inhibit DC maturation induced by LPS and diminish T cell proliferative response^27^. Also, Gringhuis et al*.*^21^ have reported that DC-SIGN signalling provoked by fucosylated ligands of *S. mansoni* plays an important role in Th2 polarization. Sensing of these ligands by DC-SIGN activates Bcl3 mediated pathway in DCs, leading to up-regulated IL-10 production coupled with abrogation of TLR-provoked proinflammatory cytokine expression (IL-6, IL-12 and IL-23). These downstream events in DCs following DC-SIGN interaction with helminth glycans have been shown to drive Th2 polarization.

Treg cells have been shown to be critical in inducing and maintaining immune tolerance^56^. They achieve their function through the release of cytokines IL-10 and TGF-β that suppress excessive immune responses. Our previous investigation revealed that DC treatment with ES L1 leads to the expansion of Tregs that suppress the proliferation of allogeneic PBMCs^14^. This study highlights the importance of DC-SIGN in the induction of higher percentage of CD4+CD25+Foxp3+ T cells, as well as IL-10 and TGF-β producing Tregs, under the influence of ES L1. Furthermore, DC-SIGN synergizes with TLR2 and TLR4 to drive T regulatory immune responses, which is expected to contribute to the maintenance of homeostasis.

In conclusion, *T. spiralis* tolerogenic DCs “signature” is initiated by the concomitant signaling of TLR2, TLR4 and DC-SIGN provoked by muscle larvae ES L1 products which is of key importance for driving a T regulatory response. An investigation to underpin the intracellular crosstalk underlying the above mentioned events is already underway. The knowledge generated from such studies is crucial for development of the effective immunotherapy to be used when Treg functions are disturbed, as it is the case with autoimmune diseases.

## Methods

### Ethics statement

ES L1 products were isolated from *Trichinella spiralis*. Adult male Wistar rats, used for maintaining of *Trichinella spiralis* strain (ISS 7564) were obtained from Military Medical Academy (MMA, Belgrade, Serbia) and were housed under standard conditions of the animal facility with access to food and water ad libitum. All procedures involving animals were approved and carried out in accordance with the guidelines and regulations by the local ethics committee of the Institute for the Application of Nuclear Energy—INEP, University of Belgrade, Serbia, Belgrade (permission date: February 23, 2017 in Belgrade, No. 04-406/5).

Samples of human peripheral blood were obtained from healthy volunteers after written informed consent from all participants in accordance with the Declaration of Helsinki and approval by the Ethical Board of the Institute for the Application of Nuclear Energy, University of Belgrade (permission date: November 11, 2019 in Belgrade, No. 02-76513). The experiments protocols involving humans were carried out in accordance with the Institutional guidelines and regulations.

### Preparation of *T. spiralis* muscle larvae excretory–secretory products (ES L1)

In short, Wistar rats were inoculated *per os* with 8500 *T. spiralis* larvae. After 2 months, larvae were recovered from animals using artificial digestion^9,58^. Isolated muscle larvae were cultured in complete Dulbecco’s modified Eagle medium (DMEM, Sigma–Aldrich) supplemented with 10 mM N-2-hydroxyehtylpiperazine-N′-2-ethanesulfonic acid (HEPES), 2 mM l-glutamine, 1 mM Na pyruvate (Sigma–Aldrich) and 50 U/ml of Penicillin–Streptomycin (Galenika, Belgrade, Serbia) under controlled conditions (37 °C, 5% CO_2_)^9^. After 18 h, culture supernatants were collected and then excretory–secretory products of the muscle larvae (ES L1) were obtained by extensive dialysis against phosphate buffered saline (PBS) and concentrated^9^. In order to remove the potential endotoxin contaminant in these products, SERVA Blue PrepProtein Endotoxin ExMicroKit (AMS Biotechnology) was used according to the manufacture’s guidelines. The quality of ES L1 was analysed using *Trichinella* ELISA test (INEP, Serbia). Endotoxin level in ES L1 products (50 µg/ml) as measured by Limulus Amoebocyte Lysate (LAL) turbidimetric test, was lower than 0.5 EU/ml (the limit provided by the US Food and Drug Administration (FDA) guidelines). Afterwards, the products were filtered through a 0.22 µm filter (Millipore) and stored at − 20 °C for later use.

### DC-SIGN-Fc binding assay

ES L1 products (5 μg/ml; 100 μl/well) in 0.05 M carbonate buffer (pH 9.5), were immobilized on microwell plates for 18 h, at 4 °C. Unadsorbed products were removed by aspiration, wells were rinsed three times with 300 μl 0.05 M PBS (pH 7.2), and then blocked with 1% BSA (200 μl/well) in 20 mM Tris–HCl buffer (pH 7.4, containing 150 mM NaCl, 1 mM CaCl_2_ and 2 mM MgCl_2_). Blank wells were only BSA-treated, i.e. without ESL1. After washing steps, serial dilutions of recombinant human DC-SIGN/CD209-Fc chimera protein (DC-SIGN-Fc, R&D systems) (0.06–4 μg/ml; 50 μl/well) were added followed by incubation for 2 h at 37 °C. Bound DC-SIGN-Fc chimera protein was detected with biotinylated mouse monoclonal anti-human IgG (Fc specific) antibody (1:150,000; 50 μl/well) (Sigma-Aldrich). The reaction was developed with TMB (3,3′,5,5′-Tetramethylbenzidine)-substrate solution and the absorbance was measured at 450 nm on a VICTOR multilabel plate reader (PerkinElmer). As a non-specific binding control, biotin-labelled anti-human IgG (Fc fragment) antibody was added to the plate without the previous incubation with DC-SIGN-Fc, and obtained OD value was subtracted from those obtained with DC-SIGN-Fc chimera protein. Binding of DC-SIGN-Fc chimera protein to mannan coated plates (20 μg/ml, 100 μl/well; Sigma-Aldrich) was used as a positive control. In order to evaluate whether DC-SIGN-Fc binding was mediated by a C-type lectin interaction, inhibition experiments using mannan (1 mg/ml), as well as the calcium-chelating agent EDTA (10 mM) were performed.

### Identification of binding of DC-SIGN to ES L1 products by Western blot

ES L1 products (25 µg/ml) were resolved on 10% separating gel with 4% stacking gel under reducing conditions, according to Laemmli^59^. Proteins were transferred onto Immobilon-P PVDF membrane and the membrane was blocked with 3% BSA in PBS (0.05 M, pH 7.2) for 2 h at room temperature. Membrane was further incubated with DC-SIGN-Fc (6.5 µg/ml in TSM, pH 7.4) for 18 h, at 4 °C, and then with biotinylated mouse monoclonal anti-human IgG (Fc specific) antibody (1:150,000 in TBS containing 0.05% BSA) for 2 h at room temperature. After washing steps, Vectastain Elite ABC reagent was added. Results were visualized with Pierce ECL Western Blotting substrate (Thermo Fisher Scientific).

### Human monocyte and T cell isolation

Peripheral blood mononuclear cells (PBMCs) from healthy blood donors were separated by gradient centrifugation on Lymphoprep Density Gradient Medium (STEMCELL Technologies). Then, CD14^+^ CD16^–^ monocytes and CD3^+^ T lymphocytes were purified from human PBMCs by using the Classical Monocyte Isolation Kit and the Pan T cell isolation kit (both from Myltenyi Biotec) according to the manufacturer’s instructions. The purity of CD14^+^ monocytes and CD3^+^ T cells, as determined by flow cytometry analysis, was consistently > 90% (data not shown).

### Cultivation and the treatment of monocyte-derived DCs

To generate immature conventional DC, purified monocytes were cultured in 24-well plate, at a density of 0.5 × 10^6^ cells per well for 6 days in CellGenix GMP DC medium (CellGenix) supplemented with 100 ng/ml of human recombinant GM-CSF and 20 ng/ml of human recombinant IL-4 (both from R&D Systems). The cells were replenished with fresh medium on day 3. To access the role of DC-SIGN on human DCs in binding ES L1 and possible consequential modulation of TLR-signaling pathway, on day 4, the cells were pre-incubated for 1 h with blocking DC-SIGN antibodies (20 µg/ml) (CD209 (DC-SIGN) purified, Beckman Coulter) alone or in combination with anti-TLR2 (10 µg/ml) (purified anti-human CD282, BioLegend) and anti-TLR4 (10 µg/ml) (purified anti-human CD284, BioLegend) blocking antibodies and then treated with ES L1 products (50 μg/ml) for the next 48 h. In some experiments, simultaneous blocking of TLR2 and TLR4 was also used as a control for DC-SIGN engagement in ES L1 effect on DCs. In order to examine whether the DC phenotype induced by ES L1 is stable, these cells were also treated with LPS (500 ng/ml, Sigma-Aldrich) for the last 24 h. The cells pre-incubated with appropriate isotype control antibodies (anti-rat IgG, e-Bioscience) before the treatment with ES L1 were used as negative controls. Also, cells cultivated only in medium were used as a negative control and those treated with LPS (500 ng/ml) as a positive control. After incubation, the cells were harvested and prepared for phenotype analyses or for functional assays with T cells.

### Allogeneic T cell-stimulatory capacity of DCs

The capacity of treated and non-treated DCs to induce T cell polarization was analysed in allogeneic stimulation assay. Allogeneic T cells were purified from peripheral blood mononuclear cells, isolated from buffy coat of healthy donors, and then immediately were co-cultivated (1 × 10^5^/well) with DCs (0.5 × 10^4^/well) in 96-well round-bottom plate in a final volume of 200 µl for 6 days. For the flow cytometric detection of intracellular cytokines, the cocultures were treated with PMA (20 ng/ml), inomycin (500 ng/ml) and monensin (3 µM) (all from Sigma-Aldrich) for the last 3 h of incubation.

Additionally, T cells were primed with DCs at a 1:50 DC/T cell ratio to assess the capacity of stimulated DCs to induce regulatory T cells (Tregs). Allogeneic T cells (1 × 10^5^/well) were plated with DCs (0.2 × 10^4^/well) in 96-well round-bottom plate, and after 3 days of incubation, 20 IU⁄mL of human recombinant IL-2 (2 ng/ml, R&D Systems) was added. The culture was incubated the next 48 h, and for the last 4 h of incubation, the co-cultures were treated with PMA/ionomycin and monensin. The expression of CD4, CD25 and FoxP3 as well as the percentage of CD4+CD25+ T cells producing cytokines IL-10 and TGF-β was determined by flow cytometry.

### Flow cytometric immunophenotyping

DCs treated as indicated were washed with PBS supplemented with 2% FCS and 0.1% Na-azide, and incubated (1 × 10^5^ cells) with panel of immunophenotyping antibodies for surface labeling: anti-CD83-FITC, anti-CD86-FITC, anti-CD40-APC, anti-CCR7-FITC, anti-CD1a-PE (BioLegend Inc., San Diego, CA), anti-CD14-FITC, anti-HLA-DR-PerCP (Myltenyi Biotec) and anti-Ig-like transcript (ILT)3-PE (R&D Systems) for 30 min at 4 °C. For intracellular staining cells were fixed using the flow cytometry fixation and permeabilization kit I (R&D Systems) and intracellular staining was performed with following antibodies: anti-TGF-β-PeCy5, anti-IL-12p40/p70-PE, and anti-IL-10-FITC (BioRad). Isotype-matched control monoclonal antibodies were used to determine non-specific background staining immunoglobulin (Ig)G1a negative control-peridinin-chlorophyll-protein complex (PerCP), IgG1 negative control-phycoerythrin (PE), IgG1 negative control-fluorescein isothiocyanate (FITC), IgG1a negative control-PECy5, IgG1 negative control-allophycocyanin (APC). Labeled cells were analysed on BD FACS LSR II flow cytometer and data were analysed using FlowJo software.

T cells were stained for surface markers with the following antibodies: anti-CD4-FITC, anti CD4-PE, anti-CD4-APC (eBioscience), anti-CD25-PeCy5 (BD Pharmigen) antibodies. After cell fixation and permeabilization, the following mAbs for intracellular staining were used: anti forkhead box Foxp3-PE, anti-TGF-β-APC, anti-IL-4-PerCP, anti- IL-10-PE (eBioscience) and anti-IL-17A-APC (BioLegend), anti-IFNγ-FITC (R&D Systems). The gates for cultivated DC and T cells were set according to their specific forward scatter (FS) and side scatter (SS) properties, thereby avoiding dead cells with low FS/SS signal. A minimum of 5000 cells was acquired per sample at a flow rate ranging between 50 and 100 cells/s. Data were acquired using a BD FACS LSR II flow cytometer, as well as Sysmex Partec Cube 6 and analysed using FlowJo software.

### Statistical analysis

One-way analysis of variance (ANOVA) was performed followed by the Bonferroni’s multiple comparison test or Tukey’s posttest, to analyse differences in means between different groups of treated cells and control groups (GraphPad Prism version 5.00 for Windows, GraphPad Software, San Diego, CA, USA). Data are presented as means ± SD, and differences were considered significant at p values of ≤ 0.05.

## Supplementary information

Supplementary Information.

## Data Availability

The datasets generated during and/or analysed during the current study are available from the corresponding author on reasonable request.
