# Supplementary material for: DC-SIGN signalling induced by Trichinella spiralis products contributes to the tolerogenic signatures of human dendritic cells
Source: Sci Rep. 2020 Nov 20;10:20283. doi: 10.1038/s41598-020-77497-x (PMC7679451; doi:10.1038/s41598-020-77497-x)
Supplement: Supplementary file 1 — Supplementary Information. [file 41598_2020_77497_MOESM1_ESM.pdf]

Supplementary Information

DC-SIGN signalling induced by *Trichinella spiralis* products contributes to the tolerogenic signatures of human dendritic cells

Jelena Cvetkovic[1*], Nataša Ilic[1], Alisa Gruden-Movsesijan[1], Sergej Tomic[1], Ninoslav Mitic[1], Elena Pinelli[2+] & Ljiljana Sofronic-Milosavljevic[1+]

[1]Institute for the Application of Nuclear Energy, University of Belgrade, Belgrade, Serbia
[2]Centre for Infectious Disease Control Netherlands, National Institute for Public Health and the Environment (RIVM), Bilthoven, Netherlands

[+]These authors have contributed equally to this work.

*Correspondence: Jelena Cvetkovic
E-mail: jelenac@inep.co.rs

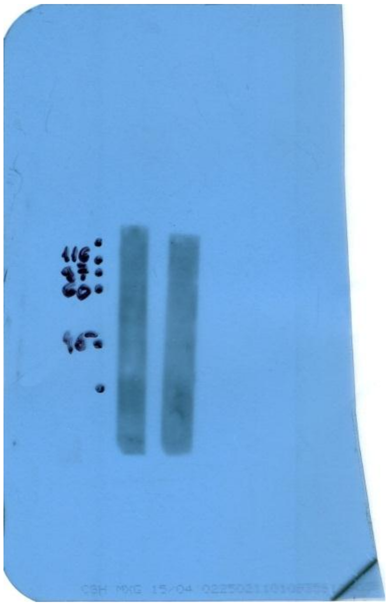

Figure S1. **Binding of DC-SIGN-Fc chimera protein to immobilized ES L1 antigens by Western blot** (the full-length blot). ES L1 antigens were resolved on 10% separating gel with 4% stacking gel. Proteins were transferred onto Immobilon-P PVDF membrane and the membrane was incubated with DC-SIGN-Fc. After incubation, the membrane was incubated with biotinylated mouse monoclonal anti-human IgG (Fc specific) antibody (first line). As a non-specific binding control, biotin-labelled anti-human IgG (Fc fragment) antibody was used without the previous incubation with DC-SIGN-Fc (negative control, the second line). Results were visualized with Pierce™ ECL Western Blotting substrate.

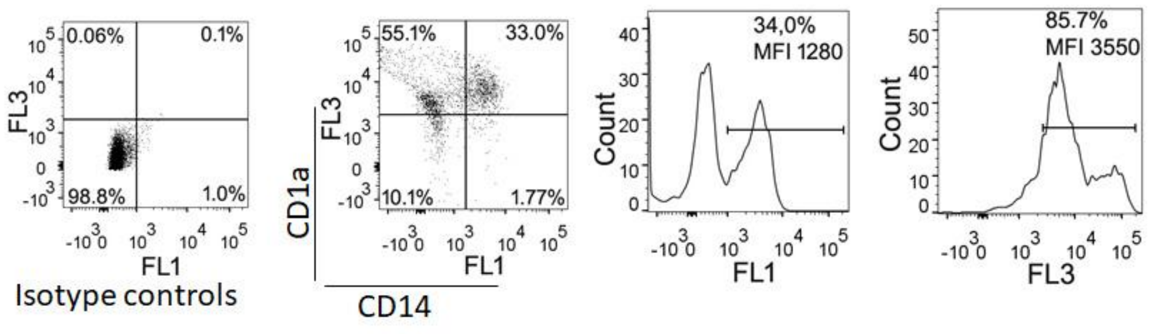

Figure S2. Differentiation of dendritic cells. Immature DCs were generated from monocytes in GM-CSF/interleukin-4 supplemented medium, during 5 days and the expression of CD14 and CD1a was analyzed by flow cytometry. Differentiation of DCs is shown as a representative plot and histograms from one out of three experiments.

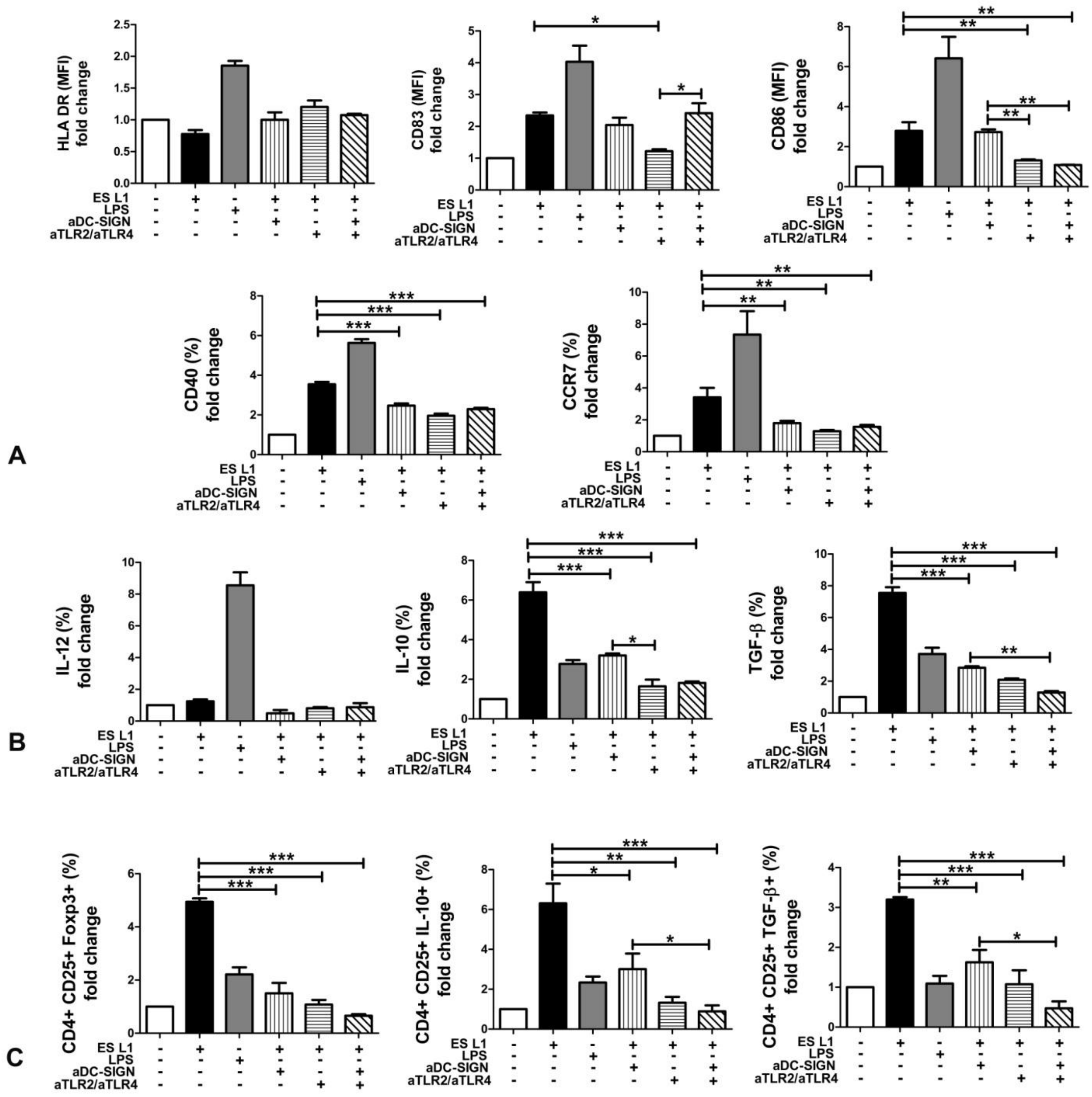

Figure S3. Impact of DC-SIGN, TLR2 and TLR4 blocking on phenotype, tolerogenic properties and functions of ES L1-treated dendritic cells (DCs). (A, B) DCs treated with specific: DC-SIGN blocking antibodies (20 ng/ml), TLR2 and TLR4 antibodies together (10 ng/ml each), or simultaneously with all 3 blocking antibodies, prior to ES L1 antigens (50 μg/ml) were washed thoroughly after 48 h of treatment and expression of DCs markers, as well as expression of cytokines within DCs was measured by flow cytometry. Non-treated cells cultured in medium only were used as a negative control (control) and LPS (200 ng/ml) treated DCs were used as a positive control for complete maturation. **(C)** Treated and non-treated DCs were cocultured with magnetic-activated cell sorting-purified allogenic T cells (Tly) (1×10^5/well) for 3 days in 1:50 DC:T cell ratio and then re-stimulated with interleukin (IL)-2 (2 ng/ml) for

another 3 days. The summarized results are shown as fold change in: **(A)** mean fluorescence intensity (MFI) or percentage (%) of markers expression on DCs ± SD from five different experiments, **(B)** % of cytokines expression in DCs as mean ± SD from five different experiments, **(C)** % of Tregs ($CD25^+FoxP3^+$ cells within $CD4^+$ T cells) and % of TGF-β or IL-10 within $CD4^+CD25^+$ T cell population, mean ± SD from five different experiments. The results of each experiment are normalized to control DCs (1). * $p < 0.05$, ** $p<0.01$, *** $p<0.005$ compared as indicated by line (One-way ANOVA with Tukey post-test).

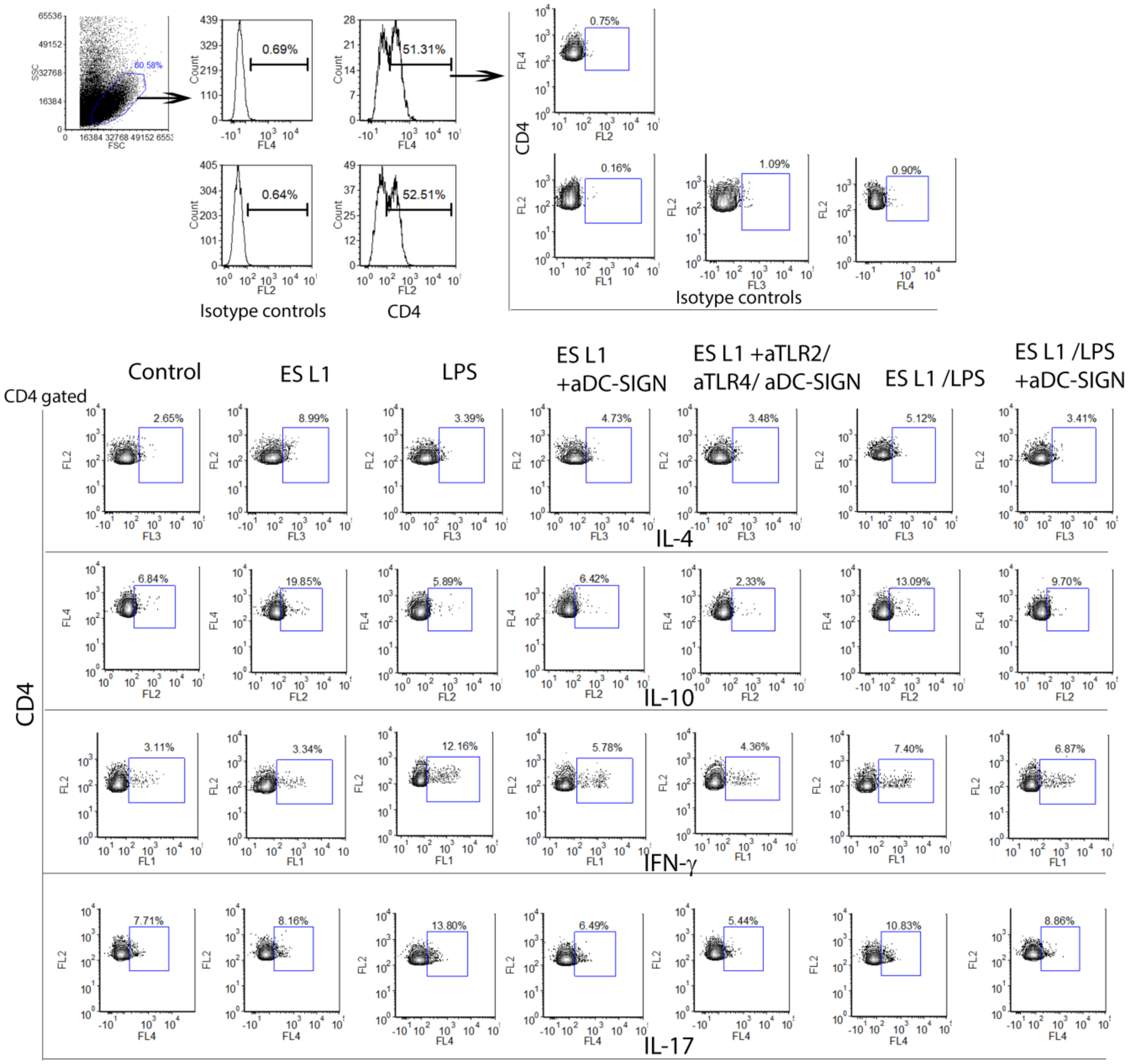

Figure S4. DC-SIGN effects on polarization of T helper cells induced by ES L1-pulsed dendritic cells (DCs). Representative results are shown for the percentage of cytokines expression measured intracellularly by flow cytometry, within the T cells cocultivated with treated or non-treated DCs (cells treated with specific DC-SIGN blocking antibodies, alone or simultaneously with TLR2 and TLR4 blocking antibodies, prior to ES L1 and/or LPS treatment).

T cells were subjected to CD4 surface staining prior to intracellular staining, and treated with PMA/Ionophore/monensin for the last 4 h.
